# Measuring Extended Water Interfacial Structures. Water Nanoclusters (∼50 nm) Involved by Ice-like II Walls (∼10 nm)

**DOI:** 10.1021/acs.langmuir.5c06652

**Published:** 2026-04-30

**Authors:** Omar Teschke, Wyllerson Evaristo Gomes, Paula Simoes Casagrande, Jose Roberto deCastro, David Mendez Soares

**Affiliations:** † Laboratório de Nanoestruturas e Interfaces, Instituto de Física, UNICAMP, 13083-859 Campinas, SP, Brazil; ‡ Faculdade de Quimica, 28101 Pontificia Universidade Catolica de Campinas, 13012-970 Campinas, SP, Brazil

## Abstract

Interfacial water layers attached to both hydrophobic and hydrophilic substrates have low dielectric permittivity. Measured structural differences between hydrophobic and hydrophilic arrangements are presented in this work. Nanosized water clusters (∼50 nm) are observed in air/water interfacial regions characterized by an ice-like structure forming walls surrounding regions with a dielectric constant ε ∼7.6. Walls are formed by an ice-like II structural arrangement. This condensed phase of water possesses an extremely small dielectric constant (ε ∼3.6) and forms wired water structures. Two nonequilibrium arrangements are then responsible for the measured spatially variable dielectric constant profiles observed in Atomic Force Microscopy.

## Graphical abstract

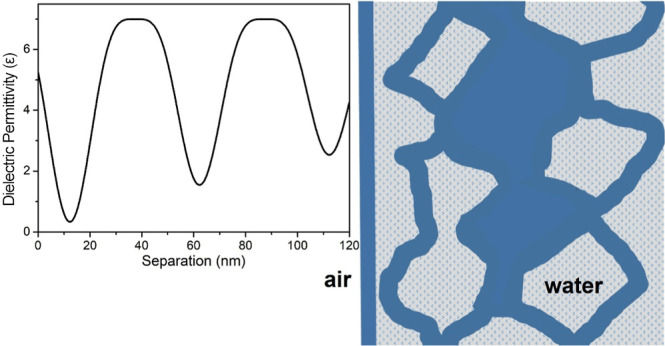

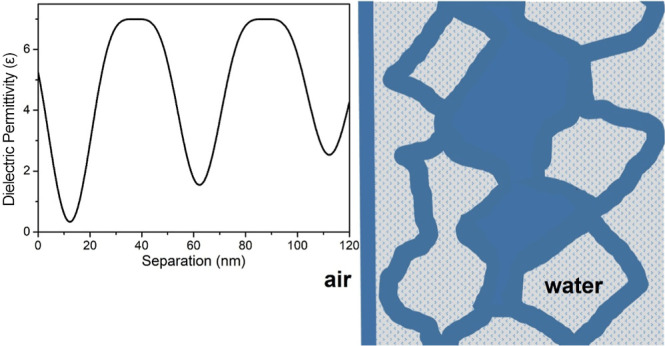

## Introduction

The molecular nature of liquid water is not yet characterized and a molecular description is still missing.^1^ Recently, experimental studies on the molecular level were reported,^2^ but a detailed model of the interfacial water structures is still absent.^3^

Here, we have, by analyzing the water structure attached to hydrophobic and hydrophilic surfaces, characterized the structured differences between them. The arrangements of the interfacial molecular regions were probed and characterized using the force versus separation curves and dielectric exchange force expression.

For interfacial water layers attached to hydrophobic and hydrophilic substrates, a measured dielectric permittivity (ε) of ε ∼5 was registered. These structural arrangements with a lower value of ε are induced at hydrophobic interfaces by the discontinuity of the water structure at the surface and at hydrophilic substrates by the surface charges.

Water structures formation around argon molecules was reported by Frank and Evans.^4^ The ordering was associated with hydrogen bond formation. Frank and Evans “icebergs” structures have lifetimes of around 10^–11^ s associated with a more structured liquid. Here, we report on interfacial water structures that have long duration and consequently can be probed by Atomic Force Microscopy (AFM). AFM tips immersed in these structures result in variable attraction or repulsion forces. These variations are associated with the water structure, in this work characterized by their dielectric constant profile distributions.

Pimentel et al.^5^ measured the mid-IR spectra of water clusters and Vernon et al.^6^ in IR predissociation experiments in supersonic beans characterized the OH stretching vibrations of gaseous water clusters. The hydrophobic molecules, individually hydrated or assembled into larger structures, show distinct patterns formation.

## Experimental Section

In this work, an atomic force microscope (TMX2000, TopoMetrix, CA, USA) operated with a silicon nitrite (Si_3_N_4_) tip (model MSCT-AUHW, Microlevers, Veeco, CA, USA) with a spring constant of ∼0.03 N/m and radius of curvature ∼5 nm was used to scan surfaces in liquid media with a few angstroms of spatial resolution.^7,8^ A special cell made from polytetrafluoroethylene (PTFE) was fabricated for observations in liquid media.^8^

Mica samples, with a 1 × 1 cm^2^ area, were cleaved with adhesive tape in air and immediately transferred to an enclosed chamber in the AFM head. The samples are several tenths of a millimeter thick and were used without any previous treatment; AFM force images show that the surface is atomically flat.

Air bubble surfaces in water were probed. A microsyringe injects a bubble into the solution and photos of the PTFE surface bubbles show that their contact angle is >90°. In order to prevent the three-phase line of air bubbles from moving laterally, they were attached to a PTFE surface. Air bubble diameters of ∼5 nm fixed to the surface result in the best reproducibility of the experimental measuring conditions. No systematic study of the effect of the size of the air bubble was made, but sizes were selected to give better measuring conditions like substrate fixation and/or observation position.

### Measuring Technique: Dielectric Exchange Force Model

The two techniques that were used in this work are the dielectric exchange force, which is our main contribution to the field, and the technique using laser-based surface-specific vibrational spectroscopy. This technique is reviewed by Bonn et al.^2^

The probed tip–air bubble interaction region is schematically shown in Figure 1. The hydrophobic water/air interface is reported as a model of ordering water where the first few layers of water molecules are highly ordered, while a normal undisturbed bulk structure is shown at some distance from the interface.

**Figure 1:**
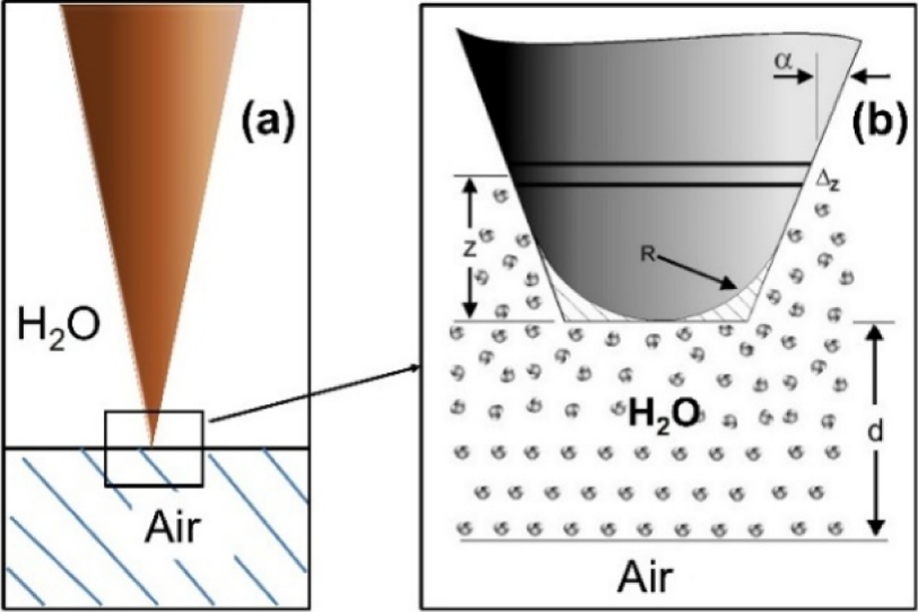
(a) Schematic diagram of the tip/air bubble configuration. (b) Conical shaped tip with a cone angle α, and a flat end with an area of π*R* ^2^ immersed in the polarization layer region; *z* is the integration variable of the elemental volume with a width Δ*z*, and *d* is the distance between the surface and end of the tip.

By using the dielectric exchange force model applied to the force versus separation measured profiles, it is possible to determine the electric field interfacial profiles. The model is based on the exchange of a nanoscopic region of the double layer by the AFM tip with a nanometer diameter. We assume that the macroscopic double layer electric field is not substantially altered by the immersion of the tip in the interfacial region, except for the exchange of water by the tip. The force on the tip still reflects the macroscopic interfacial electric field.

### Hydrophilic and Hydrophobic Substrates Variable Permittivity Profiles Determination

When a tip with a dielectric constant ε_tip_ is exchanged by a volume of a region of the double layer with dielectric constant ε_DL_, a repulsive/attractive force is detected on the tip.^9,10^ The electric field interfacial energy from the various charge distributions is probed by this measuring technique.

The spatial distribution of the dielectric permittivity at the interfacial region attached to hydrophilic substrates (mica) is calculated as follows: Curve profiles corresponding to the mica–water interfacial region were previously published.^9^ The electric displacement vector (*D*) is used to calculate the force acting on the tip^9^ by eq 1 and (*D*_0_) is determined by using the Gauss law and is assumed to have an exponential spatial dependence *D*(*z*) = *D*_0_ *e* ^–κ*z*^. The change in electric energy (*W*) resulting in force (*F*_*z*_) acting on the tip is calculated by integrating the energy expression (eq 1), where *z* is the integration variable of the trapezoidal volume, *H* is the distance between the surface and the end of the tip, and the force is given by *F*_*z*_ = −grad Δ*W*.

1 $$\Delta W=\frac{1}{2}\int_{0}^{10\kappa^{-1}-H}\left(\frac{1}{\varepsilon_{DL}\left(z\right)}-\frac{1}{\varepsilon_{tip}}\right)\frac{D^{2}\left(z\right)}{\varepsilon_{0}}\pi {\left[R+z\left(tg\alpha \right)\right]}^{2}\;dz$$

In principle, any spatial distribution for the electric displacement vector (*D*) can be used and adjusted to the measured profiles.

Observe that the tip approaches the mica surface perpendicularly, consequently the measured dielectric constant profile is the out-of-plane component. In order to obtain the in-plane profile, a scanning of points along the surface has to be made to build a surface map. The dielectric force model can be used, but extensive calculations for each probed point have to be made.

The profiles for hydrophobic substrates are calculated as follows: The electric field vector (*E*) is assumed to have an exponential spatial dependence *E*(*z*) = *E*_0_ *e* ^–κ*z*^ adjusted to fit the measured profiles at far distances from the interface. The change in the electric energy (*W*) involved in the process is calculated by integrating the energy expression of eq 2, where *z* is the integration variable of the trapezoidal volume and *H* is the distance between the surface and the end of the tip.

2 $$\Delta W=\frac{1}{2}\int_{0}^{10\kappa^{-1}-H}\left(\varepsilon_{tip}-\varepsilon_{interface}\right)\varepsilon_{0}E^{2}\left(z\right)\pi {\left[R+z\left(tg\alpha \right)\right]}^{2}\;dz$$

The force is obtained by the gradient of the energy expression *F*_*z*_ = −(∂/∂*x*) Δ*W* and it is possible to observe that in a double layer region, where ε is smaller than the ε_tip_, there is tip repulsion. The probed region is the tip scanned range in the normal direction to the surface and is typically in the nanometer range; therefore, ice-like measured profiles are observed in this range.

## Results and Discussion

### Profile of Force vs Separation Curves and Analyses

To characterize hydrophobic and hydrophilic substrates, let us initially use mica substrates. Figure 2 shows the force versus separation profile measured for a mica substrate immersed in water. The profile shows a repulsion starting ∼100 nm away from the mica substrate and an attraction at ∼15 nm. Repulsion forces are characterized by an increase of force on the tip when approaching the substrate.

**Figure 2:**
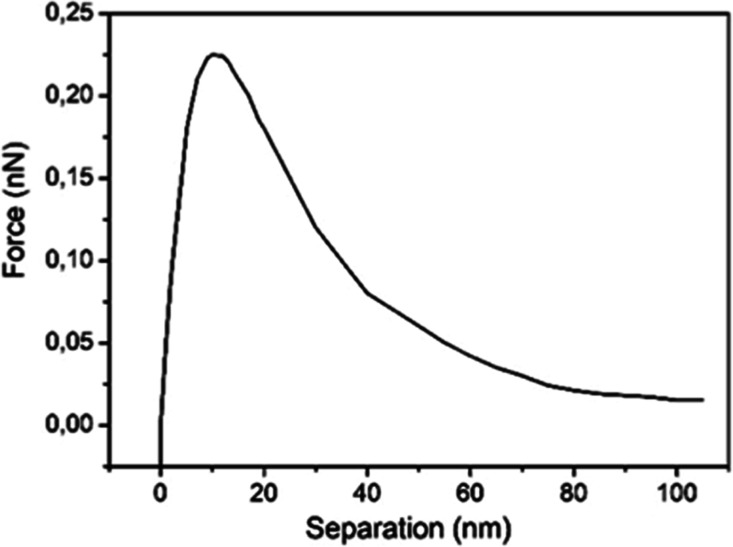
Force vs separation curves for a Si_3_N_4_ tip and a mica sample immersed in water.

Recently, the authors^9–11^ have modeled the force acting on uncharged tips when immersed in the interfacial double layer of ionic charged hydrophilic surfaces using the dielectric exchange force. A repulsion at long distances to the interface is followed by a strong attraction in the region close to it, which is associated with the alignment of water molecules within the interfacial region.

In Figure 3, the calculated dielectric permittivity profile given by eq 1, for a mica/water interface, is displayed. Attraction and repulsion regions are indicated with the corresponding value of ε.

**Figure 3:**
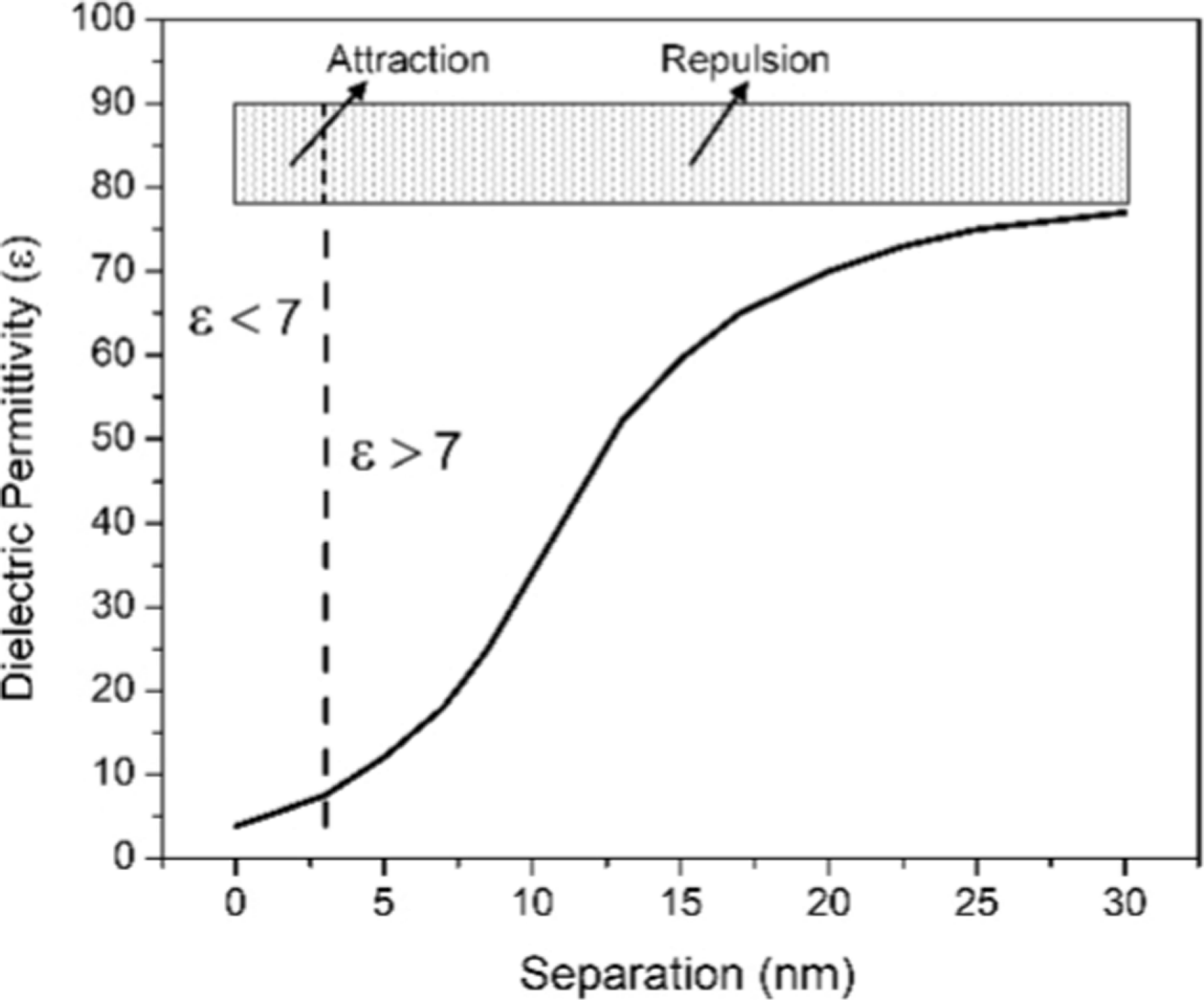
Calculated dielectric permittivity profile for a mica/water interface showing a region close to the mica surface where ε is lower than 7.6 and therefore a tip attraction for separation ≤3 nm is observed and a repulsion starting at 3 nm increasing up to ∼30 nm.

For these substrates, the force expression is given by eq 1. It is possible to observe that for a region where ε is smaller than ε = 7.6 (tip), the tip is attracted. Figure 3 shows the calculated dielectric permittivity profile for a mica/water interface at the region close to the mica surface. A tip attraction for separation ≤3 nm is observed; a repulsion is observed starting at 3 nm increasing up to ∼100 nm. Consequently, the dielectric permittivity attached to a hydrophilic substrate decreases monotonically from the bulk value ε ∼80 to ε ∼4 at the mica surface.

Figure 4 shows the force versus separation profiles observed on air/water interfaces of air bubbles deposited on PTFE substrates. The values of ε are determined by the fitting of the attraction/repulsion regions to the oscillatory values of ε in the normal direction to the surface. The force vs separation curve showing 1 step formed by repulsion/attraction regions is displayed in Figure 4a and the best fitting is obtained for ε_tip_ ∼7. Figure 4b shows 2 steps, and Figure 4c shows 3 step force vs separation curves. The best fitting in both figures is obtained for ε_tip_ ∼6.

**Figure 4:**
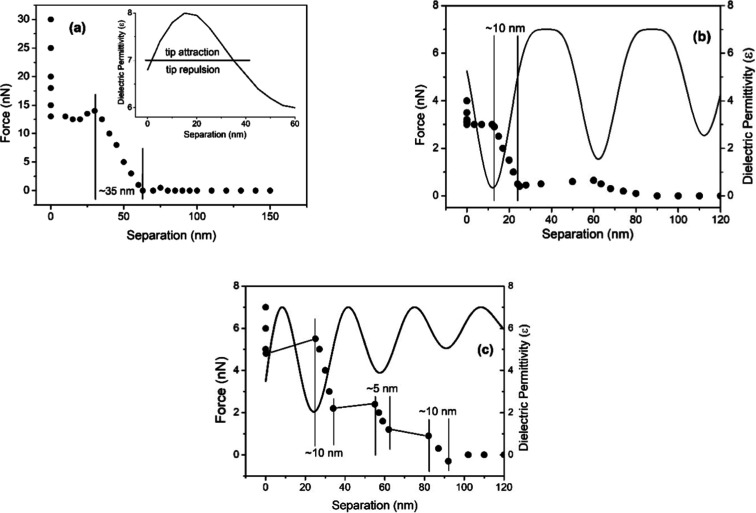
Force vs separation profiles of the interfacial regions showing clusters arrangements. The repulsion region sizes are indicated by separated vertical lines. The full line curves show the adjusted oscillating profile of the dielectric permittivity calculated using eq 2. (a) The profile shows one cluster. Observe (in the upper inset) that for the interval *x* ∼5 to ∼35 nm, the dielectric permittivity is ε_interface_ ∼7 which corresponds in eq 2 to an attraction. (b) The profile shows two clusters; for the interval *x* ∼0 to *x* ∼12 nm, there is an attraction on the tip, followed by a repulsion acting on the tip up to 24 nm. (c) The profile shows three clusters. The fast sweep in this region resulted in a nongraphical signal. The profile is similar to the almost horizontal scans shown in (a,b).

For hydrophobic profiles, the force vs separation is calculated by eq 2, and it is possible to observe that for ε <7, there is tip repulsion. Figure 4 shows various attractions and repulsion regions, and the corresponding dielectric permittivity spatial variation is the result of the best fitting of the experimental points. We propose, based on these fittings, that interfacial water shows a spatially oscillating polarization profile formed by a hydrogen-bonded network of water dipoles. This network is formed by cages of molecules with a region surrounded by a layer with a distinct arrangement of molecules, as indicated by the measured dielectric permittivity profile (see Figure 4a–c). Observe that the strong attraction at *x* ∼0, present at hydrophilic substrates, is not observed in the profile shown in these figures, but two distinct patterns of force versus separation curves for hydrophobic and hydrophilic interfaces are observed.

There is a large variation in the shape of force versus separation curves when probing air–water interfacial regions. Typical force curves include various steps in regions close to the air–water interface. Table 1 shows interfacial region profiles at different surface points. These variable profiles indicate that the interfacial regions are formed by a spatially variable structure along the normal direction to the surface. The cluster dimension column shows the size of the structure in the normal direction to the surface. The various surface arrangement sizes indicate that there is a variable profile along the surface plane.

**Table 1:** Air/Water Interfacial Profiles Formed at Air Bubbles Deposited on PTFE Substrates

surface arrangement	cluster dimension (attraction region extension) (nm)	force amplitude (nN)	surface distance (nm)
1	8.5	1.4	34
	8.5	3.3	25.5
	17	5.0	17
2	8.5	0.7	8.5
3	25	2.3	27
	2	5	2
4	25.5	7	93.5
	68	14	68
5	25	4.5	24
6	25	7	24
7	25	4	67.5
	42.5	5	42.5
8	34	3.7	76.5
	42.5	4.5	42.9

These force versus separation profiles were also measured for polycrystalline gold substrates, silicon, and other hydrophobic substrates, and similar repulsion/attraction profiles were observed.

A mosaic of the 3 regions of hydrophobic profiles is shown in Figure 5, i.e., measured force vs separation profiles association with their dielectric constant profiles. Regions with spatially variable ε forming clusters, i.e., regions with a value of ε surrounded by regions with a smaller value of ε ∼3.6, are shown. This arrangement characterizes the extended air–water interfacial regions.

**Figure 5:**
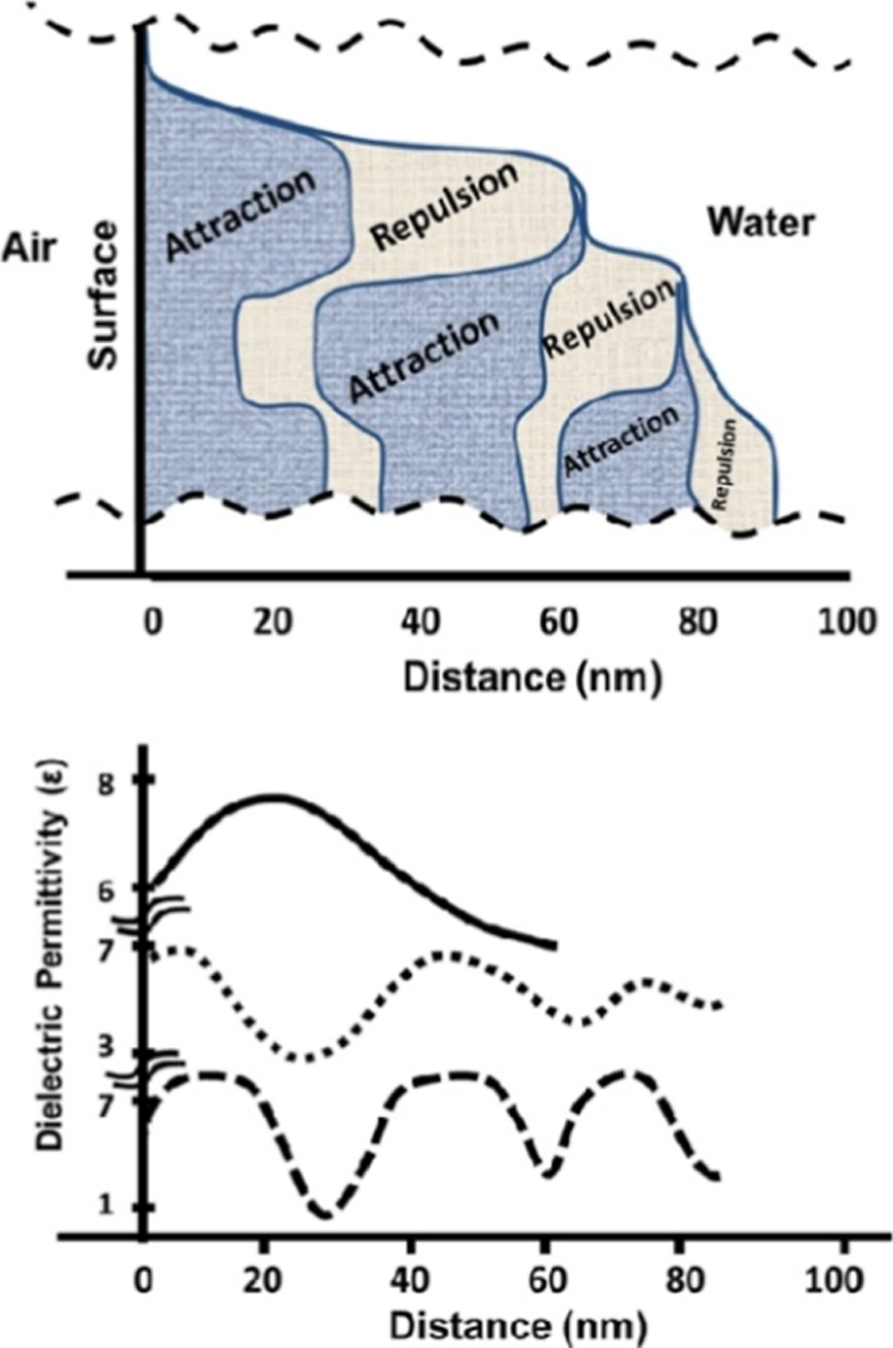
Schematic diagram of repulsion/attraction profiles shown in Figure 4 combined forming the extended profile of the interfacial region (top). At the lower part, 3 attraction/repulsion steps; at the middle, 2; and at the top, 1 attraction/repulsion step. The dielectric permittivity spatial profile corresponding to each region is shown at the bottom.

The measured dielectric permittivity profiles^11^ show a value of 3.5 ± 0.5 at the water interface. This value is calculated using eq 2 and implies a fitting to the measured curve. We believe this deviation of ±0.5 is associated with the fitting of curves.

### Cluster Attached to Nafion Surfaces

Water cluster formation was previously observed in water interfacial regions attached to Nafion surfaces.^12^ The Nafion surface observed in air shows a surface fibrillary structure formed by hydrophilic and hydrophobic regions. Our study was focused on the structure of hydrophilic domains.^13–15^ Studies of small-angle X-ray and small-angle neutron scattering resulted in a new model comprising fibrillary aggregates of a hydrophobic polymer with hydrophilic side chains that protrude radially outward.^16–18^ The water structure attached to these surface arrangements was observed by their dielectric profiles; a mixture of hydrophobic and hydrophilic sites is formed.^19^

Strong attractions between water and hydrophilic units tend to localize water molecules to specific locations and thereby limit fluctuations, which is important for the self-assembly in water of amphiphilic molecules containing hydrophobic as well as hydrophilic components. Such solutions form an array of mesoscopic assemblies that are at least partly stabilized by hydrophobic forces.^20,21^ The principles that hold for purely hydrophobic solutes also apply to molecules containing some hydrophilic units, but additional entropic effects arise because of these molecular configurations. The simplest example of an amphiphilic assembly is the Nafion interfacial region.

The segregation of hydrophobic and hydrophilic phases extends over distances that are large compared with the distances over which molecules affect one another in a homogeneous liquid. This segregation determines the size of the Nafion surface cluster arrangement.

### Large Water Cluster Formation Observed at Extended Water Surfaces

A hydrogen-bonded polyhedral clathrate cage is supposed to form when nonpolar molecules are present around the solvent molecules. Then the structure of water surrounding small hydrocarbons presents the so-called clathrate-like water structure close to the small hydrophobic entity. Hydrophobic interaction seems to cause water clustering attached to large hydrophobic surfaces, while remnants of the clathrate structure persist in the liquid only near a small hydrophobic particle.^22^ Different characteristics of the hydrophobicity are manifested depending on the large regions or small molecular units involved or a combination of both. The physical basis for understanding hydrophobic effects was expressed by Stillinger^23^ more than 30 years ago.

The difference in the characteristics of hydration of small solutes compared with flat hydrophobic surfaces is related to the hydrogen bond network. At extended interfaces, there is the ability of water molecules to retain their hydrogen bond network in the interfacial region.^24^ Electric polarization forces should weaken the hydrogen bond network near the wall. Many-body dispersion forces are expected to play an important role in determining the surface tension of water, the liquid structure, and hydrophobic effects near surfaces.^24^

In Figure 5 a schematic diagram of a water interfacial arrangement where regions with 3 clusters in contact with regions with 2 clusters and at the top a region with 1 cluster is shown. Table 1 lists various surface cluster arrangement dimensions; arrangement 1 is formed by 3 clusters that extend 34 nm away from the surface. Arrangements with 2 clusters are the most common observed attached to our probed regions.

Previous works have reported the presence of nanosized water clusters in the interfacial regions.^25,26^ The dielectric constant of confined water and interfaces was also previously reported.^27–29^ Here, we have investigated structures attached to extended hydrophobic surfaces and characterized interfacial water structures by their dielectric permittivity (ε) profiles.

### Wired Water Walls Surrounding Water Clusters

Robinson et al.^24^ characterize the properties of ice by their dielectric constant values (see Table 4.4 in page 106). Ice Ih, II, III, V, VI, and IX are shown to have distinct dielectric constants values. Here, we report a measured value of ε ∼3.6 which corresponds to the value of ice II. Since ice Ih is the most common form, it may transform into ice II, which is the value observed in the interfacial region.

Observed interfacial structures show a central part involved by outer layers (walls) in Figures 3 and 5. Strongly hydrogen-bonded water molecules form these water walls surrounding the water clusters. These walls are characterized by their measured dielectric constant ε ∼3.6. The thicknesses of these ice-like II walls are typically ∼10 nm. Wider structures reaching ∼35 nm are also shown.

Table 2 shows the dielectric constant profiles for the three arrangements. The indicated width is the dimension of the structures in the normal direction to the interfacial plane. Layers with constant dielectric values of 3 and 7 are shown. The measured value of 3 corresponds to an ice-like II structure.

**Table 2:** Structure Width (nm) and Dielectric Constant for Three Distinct Arrangements

ε	arrangement I width (nm)
7	12
3	13
7	35
3	20

ε	arrangement II width (nm)
8	30
3	33

ε	arrangement III width (nm)
7	25
3	9
7	21
3	7
7	20
3	10

Another piece of evidence for the presence of an ice-like structure in the interfacial region came from the Raman spectra measured at the floating water bridge interfacial regions. The Raman spectrum was registered at floating water bridges because in this arrangement, there is an increased number of protons that are transported along the interface induced by an applied longitudinal electric field, which results in a large enough number to be detected by confocal Raman microscopy.

The hydrogen bond network Raman spectrum in interfacial water was recently measured.^30^ The modes were probed at an applied voltage of 3 kV (*E* = ∼10^6^ V/m). The local structures probed comprise different configurations. The Raman spectra of interfacial water are shown in Table 3.

**Table 3:** Interfacial Water Confocal Raman Microscopy Measured Peaks (cm^–1^) Compared to Ice II in Three Regions: Vibrational Modes (*v*_1_, *v*_2_, and *v*_3_), Librational Modes (*v*_L_), and Translational Modes (*v*_T_) [Ref 24, Page 103, Table 4.2]

Raman line	*v*_1_	*v*_2_	*v*_3_	*v*_L_	*v*_T_
interfacial water	3190	1650	3320	860	170
ice II	3194	1690	3314	800	151

Observe that there are small differences between their spectra; for this reason, we call the observed structure ice-like II.

The low value of the dielectric permittivity ε ∼3 was measured for both hydrophobic and hydrophilic interfacial regions,^11,31^ but the water clusters were observed only at hydrophobic interfacial regions. This water cluster structural pattern at hydrophilic surfaces is probably erased by the uniform electric field generated by the substrate surface charge distribution.

Previous work^32^ looked for conductive wires for proton transport, but this structure is not observed here. We claim that the profiles shown in Table 3 are associated with surfaces and not wires, as shown schematically in Figure 6.

**Figure 6:**
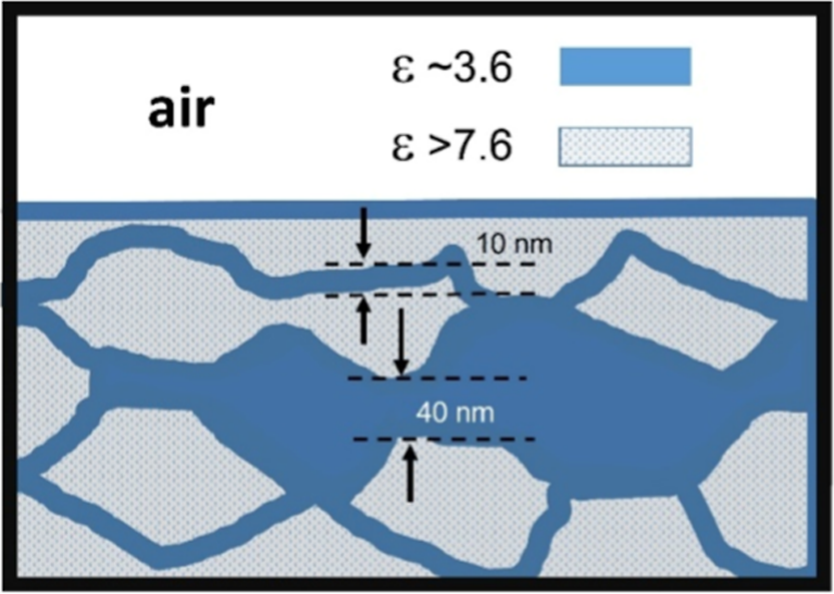
Schematic diagram of the pattern observed at an interfacial air–water region. An interconnected arrangement of proton conductive (ε ∼3.6) layers surrounding the water cluster with ε >7.6.

Water wired surfaces were detected using an atomic force microscope, which gives the structural arrangement shape and form by measuring the dielectric constant spatial variation profiles. The structure with ε ≈3.66 corresponds to ice-like II. AFM is then promising to unravel hydrogen-bonding complication configurations in water in biological systems.

Ice Ih and ice II are ice polymorphous with the defined crystalline structure as shown in Robinson’s work, page 103, Table 4.2. The observed interfacial structure has a Raman line spectrum similar to ice II and for this reason is called ice-like II.

The observed AFM interfacial profile suggests the formation of various irregular clusters surrounded by walls extending along the air/water interface. These interconnected walls formed by ice-like II form an irregular arrangement that varies locally. Molecular dynamic simulations^34–39^ could be used to describe the observed structures, since the variation in the dielectric permittivity response across nonpolar and polar liquid interfaces can be modeled by molecular dynamics simulations.

In order to validate the observed structures at the atomic scale (∼few angstroms), a distinct probing technique has to be used because AFM probing liquid structures detected an electric field pattern of the size of the tip diameter, which is substantially larger (∼few nanometers) than the atomic separation.

AFM in principle could be employed to probe the orientation of water molecules since the orientation of the molecules generates a distinct electric field configuration, but a new scheme, i.e., probe-water structure configuration, has to be devised in order to observe this structural arrangement.

A hydrogen bond is an electrostatic attraction between molecules mediated by a positively charged hydrogen atom. This relatively weak bond was first theoretically described in 1920,^33^ and corresponds to the fundamental binding force in water, explaining many of these liquids unique properties, such as its high surface tension and unique attributes as a solvent. Here, we have shown that the hydrogen-bonded network connecting H_2_O molecules in water can result in the formation of clusters involved by walls, resulting in surfaces interconnected arrangements, and AFM images provided evidence that has been lacking.

## Conclusions

To characterize hydrophobic or hydrophilic interfaces attached to substrates, AFM force vs separation profiles were measured. The force versus separation profile for a mica surface immersed in water shows a repulsion that extends up to ∼10 nm away from the surface followed by an attraction attached to the surface. This pattern is shown in Figures 2 and 3. Hydrophobic surfaces display force versus separation profiles characterized by oscillating force profiles as shown in Figure 4a–c. Then a spatial variable molecular orientational ordering is observed near an air/water interface characterized by their oscillating dielectric constant measured values. These molecular orientational orderings form clustered structures (see Figure 4a–c). A central region with ε ∼7.6 is involved by a structure with ε ∼3.6 which corresponds to the structure of ice-like II, which is formed by open hexagonal channels of ice Ih that has collapsed to a more compact structure.^21^ The interfacial water structure is then formed by an ice polymorph where ice-like II is present forming the outer layer involving clusters with dimensions as large as 50 nm with a value of ε >7. The walls surrounding the cluster form the interconnection of surface arrangements that transfer protons along the interfacial region.
